# Sociodemographic determinants of mobility decline among community-dwelling older adults: findings from the Canadian longitudinal study on ageing

**DOI:** 10.1186/s12877-024-05582-1

**Published:** 2024-11-27

**Authors:** Ogochukwu Kelechi Onyeso, Chiedozie James Alumona, Adesola Christiana Odole, Janice Victor, Jon Doan, Oluwagbohunmi A. Awosoga

**Affiliations:** 1 Faculty of Health Sciences, University of Lethbridge, Lethbridge, AB Canada; 2 Department of Physiotherapy, Faculty of Clinical Sciences, College of Medicine, University of Ibadan, Ibadan, Oyo Nigeria; 3 Department of Kinesiology, Faculty of Arts and Science, University of Lethbridge, Lethbridge, AB Canada

**Keywords:** CLSA, Four-meter walk test, Gerontology, Healthy ageing, Independent living, Life course, Mobility limitation, Social determinants of health, Timed-up and go test

## Abstract

**Background:**

Mobility is fundamental to healthy ageing and quality of life. Mobility decline has been associated with functional impairment, falls, disability, dependency, and death among older adults. We explored the sociodemographic determinants of mobility decline among community-dwelling older Canadians.

**Methods:**

This study was a secondary analysis of a six-year follow-up of the Canadian Longitudinal Study on Ageing (CLSA). Our analysis was based on 3882 community-dwelling older adults 65 years or older whose mobility was measured using timed-up and go (TUG) and 4-meter walk (4MWT) tests at baseline and follow-ups 1 and 2 after three- and six-year intervals, respectively. We analysed the cross-sectional and longitudinal association, main and interaction effects of the participants’ sociodemographic characteristics on mobility decline using chi-square, Pearson’s correlation, mixed-design repeated measures ANOVA, and bivariate and multivariate linear regression tests.

**Results:**

At baseline, 52% of the participants were female, 70.4% were married, and the average age was 68.82 **±** 2.78 years. Mean TUG and 4MWT scores were 9.59 **±** 1.98 s and 4.29 **±** 0.95 s, respectively. There was a strong positive longitudinal correlation between TUG and 4MWT (*r* = 0.65 to 0.75, *p* < 0.001), indicating concurrent validity of 4MWT. The multivariate linear regression (for TUG) showed that older age (β = 0.088, *p* < 0.001), being a female (β=-0.035, *p* < 0.001), retired (β=-0.058, *p* < 0.001), Canadian born (β=-0.046, *p* < 0.001), non-Caucasian (β=-0.063, *p* < 0.001), tenant (β = 0.050, *p* < 0.001), having no spouse/partner (β=-0.057, *p* < 0.001), household income of $50,000-$99,999 (β = 0.039, *p* < 0.001), wealth/investment lower than $50,000 (β=-0.089, *p* < 0.001), lower social status (β=-0.018,*p* = 0.025), secondary education and below (β = 0.043, *p* < 0.001), and living in certain provinces compared to others, were significant predictors of a six-year mobility decline.

**Conclusion:**

Our study underscored the impact of modifiable and non-modifiable sociodemographic determinants of mobility trajectory. There is a need for nuanced ageing policies that support mobility in older adults, considering sociodemographic inequalities through equitable resource distribution, including people of lower socioeconomic backgrounds.

## Background

Mobility has been defined in various ways and contexts [1], typically emphasising an individual’s ability to move around safely and independently, with or without the use of an assistive device [2–6]. In this paper, mobility is conceptualised as an objective measure of ambulation, specifically an individual’s movement from one point to another, with or without a walking aid. Maintaining mobility is crucial for older adults as it promotes healthy and successful ageing by enhancing physical health, social engagement, and overall well-being [7, 8].

Conversely, mobility limitations – the inability to achieve full mobility potential have far-reaching consequences [9]. These include early physical disability and institutionalisation [10], frequent falls and injuries [5], sedentary behaviour and dependency [11, 12], depression [13], social isolation [14], reduced quality of life [15], and death among older adults [10]. As the global population of older adults continues to rise [16], age-related mobility decline has become a relevant subject worldwide [17]. In Canada, approximately 20.6% of community-dwelling older adults (65 years and older) have mobility limitations, a trend that increases with age [18]. The Canadian older adult population, which was 7.3 million in 2022 [19], is projected to reach 10.9 million by 2036 [20]. Consequently, mobility decline can be predicted to exert a significant burden on Canadian social, economic, and healthcare systems [11].

The dire implications of mobility limitations in the ageing population have prompted stakeholders including older adults, caregivers, geriatricians, researchers, and policymakers, to explore modifiable sociodemographic determinants of mobility decline [17]. While many studies have focused on biomedical factors affecting mobility in older adults, the role of sociodemographic determinants is equally crucial for promoting healthy ageing [8, 9, 12, 20]. However, there is a paucity of research explicitly aimed at estimating the influence of multiple sociodemographic factors on mobility decline among older adults [21, 22]. For instance, the influence of marital status, area of residence, income, occupation, religion, homeownership, and social status on mobility trajectories remains understudied.

Understanding the modifiable sociodemographic determinants of mobility decline is essential for preventing total disability in the ageing population [17]. Such knowledge can inform advocacy efforts for personal characteristic modifications, guide clinical practices, and shape policy design and implementation [7]. To gain insights into these determinants, we analysed the Canadian Longitudinal Study on Aging (CLSA) dataset [23]. The CLSA contains objective mobility measures, such as the 4-metre walk (4MWT) and timed-up and go (TUG) tests [6, 24], and sociodemographic variables, including age, sex, education, rural/urban residence, marital status, religion, ethnicity, culture, income, employment type, retirement, house type and ownership, and wealth [23]. The longitudinal design and large sample size of the CLSA also enabled the concurrent validation of the normal-paced 4MWT using the TUG test as a criterion. This addresses a critical gap in the literature, as the few existing validation studies on non-disease-specific, community-dwelling older adult populations have been limited by cross-sectional designs and small sample sizes [25, 26].

The overarching aim of our study was to estimate the longitudinal association between older Canadians’ mobility decline and their sociodemographic characteristics using the CLSA’s baseline, follow-up 1 (FU1), and follow-up 2 (FU2) data cycles. We hypothesised that there would be a significant (i) difference in the sociodemographic distribution of the participants at baseline, FU1, and FU2, (ii) correlation between TUG and 4MWT scores at baseline, FU1, and FU2, (iii) mean differences in the participants’ TUG and 4MWT scores at baseline, FU1, and FU2 across categories of sociodemographic factors, (iv) zero-order association between TUG and 4MWT scores and sociodemographic factors at baseline, FU1, and FU2, and (v) multivariate longitudinal association between TUG and 4MWT scores and sociodemographic factors.

## Methods

### Data source

The study was a secondary data analysis of CLSA’s comprehensive cohort baseline, FU1 and FU2. The CLSA is an ongoing large national bilingual (English and French) longitudinal study that commenced in 2011 with a comprehensive cohort of 30,097 aged 45 to 85 years [23]. Sociodemographic interviews, biospecimen sampling, and physical measures, including the 4MWT and TUG, were collected in person from this comprehensive cohort at designated data collection centres in their respective cities. The CLSA participants will be followed up in three-year cycles for at least 20 years [27].

We selected 3,882 participants who were 65 years and older at baseline, participated in the three data cycles (baseline, FU1 and FU2) at three-year intervals, and never lived in a long-term care facility throughout the study period. Therefore, 113 persons who transited to long-term care facilities during the follow-ups were excluded. The overall CLSA’s participant inclusion and exclusion criteria, study protocol, procedures, consent, and ethics approval have been extensively discussed in previous publications [23, 27]. The original CLSA protocol was reviewed and approved by 13 university-based ethics committees across Canada [27]. Furthermore, the Health Research Ethics Board of the University of Alberta approved the study protocol for this CLSA secondary analysis (reference number: Pro00129371). More information about the CLSA can be found at www.clsa-elcv.ca.

### Mobility outcomes

The outcomes in this study were time (s) of completion of TUG and 4MWT measured with a stopwatch and meter rule [27]. The TUG, developed in Canada by Podsiadlo and Richardson [28], has become the most popular comprehensive mobility assessment tool for older adults because it measures functional mobility comprising of gait, balance, and transfer [6]. Moreover, it has good psychometric and clinimetric properties among community-dwelling older adults, including a high interclass correlation reliability (*r* = 0.99) [28] and a strong construct validity with gait speed test (*r* = 0.75) [29]. Similarly, the 4MWT has a high interclass correlation reliability (*r* = 0.96 to 0.98) and strong concurrent validity with the 10-meter walk test (*r* = 0.93) [25].

### Explanatory variables

The explanatory variables were sociodemographic variables identified in the CLSA, including age, sex, marital status, country of birth, ethnic and cultural identities, province, area of residence, education, occupation, total household income (THI), wealth (value of total savings and investments), homeownership, home type, religious affiliation, retirement status, and social status. Social status was measured using the MacArthur Scale of Subjective Social Status [30], by asking individuals to rank themselves on a 10-rung ladder, indicating where they stand in relation to others in their community.

### Covariates

The regression models were controlled for covariates, such as smoking, alcoholism, chronic disease status, and self-reported general health [9].

### Variable description

Age (years), TUG (s), and 4-MWT (s) were scale variables. The ordinal continuous variables were social status ladder (1–10) and alcohol frequency (1 = never, 2 = less than once monthly, 3 = once monthly, 4 = twice or thrice monthly, 5 = once weekly, 6 = twice or thrice weekly, 7 = four to five times weekly, and 8 = almost daily). The categorical variables were sex (male/ female), marital status (have partner/ do not have a partner), cultural identities (Caucasian/ non-Caucasian), country of birth (Canada/ OECD excluding Canada/ non-OECD), ethnicity (Canada/ French/ English/ Others), area of residence (rural/ urban), province (Alberta/ British Columbia/ Manitoba/ Newfoundland & Labrador/ Nova Scotia/ Ontario/ Quebec), education (secondary and below/ above secondary), occupation (manual/ non-manual), THI (<$50,000/ $50,000–$99,999/ $100,000 and above), wealth (<$50,000/ $50,000–<$100,000/ $100,000–<$1 M/ $1 M and above), home ownership (own/ rent or not own), home type (house/ apartment), religious affiliation (no/ yes), retirement (retired/ partly or not retired), social status (≤ 3-low/ 4 to 6-middle/ ≥7-high), smoked 100 cigarettes ever (no/ yes), and chronic disease status (no/ yes), general health rating (poor/ good).

### Data analysis

The data were analysed using the Statistical Package for Social Sciences (SPSS) version 28. All analysis was completed using the weighted dataset. Participants’ sociodemographic and mobility outcomes were summarised using descriptive statistics: frequency, percentage, mean, and standard deviation.

Before the inferential analysis, the data were tested for assumptions of the statistical tool. Continuous variables were tested for univariate and multivariate outliers using a standardised Z-score > ± 3.29 and Mahalanobis-distance approaches [31, 32]. Normality, sphericity, homogeneity of variance, and linearity were determined through Kolmogorov–Smirnov, Mauchly’s, and Levene’s tests and Q-Q plot, respectively [31, 32]. Multicollinearity was read off the regression output via variance inflation factor < 4. When multicollinearity occurs the least important of the affected is dropped from the model [32]. The TUG and 4MWT scores were log-transformed to achieve normality.

Hypothesis I was tested using Pearson’s chi-square test (χ^2^). Hypothesis II was tested using repeated measures mixed-design ANOVA (*F*), with Greenhouse-Geisser correction reported where Mauchly’s test of sphericity was violated. The post hoc pairwise comparisons were Bonferroni adjusted, and the Games-Howell test was applied where Levene’s test of homogeneity of variance was violated. Hypothesis III was tested using a bivariate linear regression, with the standardised regression coefficient (β) reported. Hypothesis IV was tested using simultaneous entry multivariate linear regression, with the standardised coefficient (β) reported. Hypothesis V was tested using Pearson’s product-moment correlation coefficient (*r*). The alpha level was set at 0.05 for all the inferential statistics.

## Results

### Sociodemographic characteristics

A total of 3882 participants were included in the analyses, and 52.4% were females. The participants’ mean age (years) **±** SD was 68.88 **±** 2.80 at baseline, 71.81 **±** 2.81 at FU1, and 74.65 **±** 2.83 at FU2. The TUG scores were 9.70 **±** 2.01s at baseline, 10.26 **±** 2.25s at FU1, and 10.90 **±** 2.50s at FU2. The 4MWT scores were 4.37 **±** 1.05s at baseline, 4.50 **±** 1.06s at FU1, and 4.63 **±** 1.03s at FU2.

At baseline, 0.3% could not walk without aid, 0.3% at FU1, and 0.8% at FU2. On a ten-step social status ladder (1–10), participants’ mean self-rating was 6.17 **±** 1.88 at baseline and 6.53 **±** 1.87 at FU1; the data was not collected at FU2. Other sociodemographic characteristics collected at baseline were language of data collection (English = 75.6%, French = 24.4%), cultural identity (Caucasian = 96.8%, non-Caucasian = 3.2%), ethnicity (Canada = 34.4%, French = 10.5%, English = 26.9%, others = 28.2%), country of birth (Canada = 79.7%, OECD excluding Canada = 16.8%, non-OECD = 3.5%), religious affiliation (no = 19.2%, yes = 80.8%), and education level (secondary or less = 33.7%, above secondary 66.3%). Between baseline and FU1, 3.9% of the participants gained education, 12.9% became more religious, 6.7% less religious, 5.9% changed marital status, while between FU1 and FU2, 2.0% gained education, 11.2% became more religious, 7.2% less religious, 6.1% changed marital status. Table 1 shows no significant difference in participant distribution across the province of residence, occupation type, sex, wealth, and household income categories across the cycles.

**Table 1:** Participants’ sociodemographic characteristics (*n* = 3882)

Parameters	Percentage (%)			df	χ2-statistic	*p*-value
	Baseline	FU1	FU2			
**Sex**				4	8.047	0.090
Female	52.4	52.4	52.2			
Male	47.6	47.6	47.7			
Others	0.0	0.0	0.1			
**Marital Status**				**8**	**30.122**	< 0.001*
Single	4.5	4.7	4.5			
Married/ Common law	76.5	74.7	71.5			
Widowed	8.9	11.2	14.5			
Divorced/ Separated	10.1	9.4	9.5			
**Province**				**12**	**0.236**	**1.000**
Alberta	5.7	5.7	5.7			
British Columbia	24.0	24.1	24.1			
Manitoba	8.9	8.9	8.9			
Newfoundland/ Labrador	3.1	3.1	3.1			
Nova Scotia	7.6	7.6	7.6			
Ontario	25.4	25.3	25.3			
Quebec	25.3	25.3	25.3			
**Area of residence**				**2**	**26.867**	< 0.001*
Rural	10.0	6.4	6.2			
Non-rural	90.0	93.6	93.8			
**Occupation**				**2**	**1.663**	**0.435**
Manual	16.2	13.7	17.6			
Non-manual	83.8	86.3	82.4			
**Retirement status**				**2**	**358.740**	< 0.001*
Retired	72.3	83.0	88.5			
Partly retried/ Not retired	27.7	17.0	11.5			
**Home**				**4**	**43.254**	< 0.001*
House (semi-/detached)	81.4	78.5	75.5			
Apartment or condominium	18.3	20.4	23.1			
Others	0.4	1.1	1.4			
**House ownership**				**2**	**25.173**	< 0.001*
Own	86.6	85.3	82.3			
Rent/ Not own	13.4	14.7	17.7			
**Total Household Income**				**8**	**5.926**	**0.655**
Less than $20,000	5.9	5.2	5.2			
$20,000 - $49,999	33.2	34.7	34.5			
$50,000 - $99,999	42.0	41.4	40.5			
$100,000 - $149,999	12.2	12.3	13.3			
$150,000 and above	6.7	6.4	6.5			
**Wealth (investments)**				**6**	**7.614**	**0.268**
Less than $50,000	23.6	24.9	25.7			
$50,000 - <$99,999	16.1	16.7	15.6			
$100,000 - <$999,99	49.9	49.2	48.2			
$1 M and above	10.4	9.2	10.5			

Source: weighted Canadian Longitudinal Study on Ageing dataset. * = Chi-square is significant at *p* < 0.05. FU1 = follow up (1) FU2 = follow up (2) *N* ≠ 3882 in variables with missing data. The percentage was calculated with valid cases

### Bivariate analysis

Pearson correlation analysis was used to determine the concurrent validity of TUG and 4MWT. The coefficient showed a strong positive correlation between TUG and 4MWT scores at baseline (*r* = 0.65, *p* < 0.001), FU1 (*r* = 0.74, *p* < 0.001), and FU2 (*r* = 0.75, *p* < 0.001).

For the regression analyses, it is important to note that TUG and 4MWT scores were time (seconds) taken to complete a fixed distance, therefore, a lower score implies better mobility. The data was coded such that when a reference category has a negative coefficient the non-referenced category has a better mobility. The bivariate regression analysis showed a significant association between mobility outcomes and non-modifiable sociodemographic variables, such that increasing age led to significant mobility decline at baseline (TUG β = 0.12, *p* < 0.001; 4MWT β = 0.09, *p* < 0.001) and all follow-ups (Table 2). Sex at birth correlated significantly with mobility decline such that males have lower TUG (β= -0.04, *p* = 0.021) and 4MWT scores (β= -0.11, *p* < 0.001) than women at baseline and better mobility in all the cycles. Moreover, country of birth, ethnicity, and cultural identity had a significant association with TUG and 4MWT scores at baseline (Table 2).

**Table 2:** Bivariate regression (zero-order correlation) between sociodemographic factors and mobility at baseline and follow-ups

Variable	Baseline TUG		Baseline 4MWT		FU1 TUG		FU1 4MWT		FU2 TUG		FU2 4MWT	
	β-statistic	*p*-value	β-statistic	*p*-value	β-statistic	*p*-value	β-statistic	*p*-value	β-statistic	*p*-value	β-statistic	*p*-value
***Non-modifiable factors***												
**Age** (ref: increase in years)	0.12	< 0.001*	0.09	< 0.001*	0.18	< 0.001*	0.14	< 0.001*	0.17	< 0.001*	0.16	< 0.001*
**Sex at birth** (ref: female)	-0.04	0.021*	-0.11	< 0.001*	-0.05	0.003*	-0.13	< 0.001*	-0.06	0.006*	-0.12	< 0.001*
**Country of birth‡**												
Canada	0.07	< 0.001*	0.06	0.003*	0.04	0.021	0.03	0.082	0.03	0.273	0.06	0.010*
OECD excluding Canada	-0.07	< 0.001*	-0.06	0.001*	-0.05	0.014*	-0.04	0.057	-0.03	0.164	-0.06	0.006*
Non-OECD	-0.01	0.819	0.00	0.986	-0.01	0.942	0.01	0.951	0.01	0.674	-0.01	0.912
**Ethnicity‡**												
Canada	0.05	0.005*	0.04	0.032*	-0.01	0.510	-0.01	0.913	-0.03	0.152	-0.01	0.580
French	0.02	0.267	0.03	0.098	0.03	0.167	0.03	0.181	0.03	0.279	0.02	0.365
English	-0.07	< 0.001*	-0.07	< 0.001*	-0.04	0.027*	-0.06	0.002*	-0.02	0.332	-0.04	0.127
Others	-0.01	0.814	0.01	0.958	0.04	0.053	0.04	0.027*	0.04	0.077	0.04	0.134
**Cultural identity‡** (ref: non-Caucasian)	-0.04	0.036*	-0.03	0.139	-0.04	0.038*	-0.05	0.008*	-0.01	0.852	-0.03	0.183
***Modifiable factors***												
**Marital status** (ref: has no partner)	-0.08	< 0.001*	-0.09	< 0.001*	-0.10	< 0.001*	-0.10	< 0.001*	-0.11	< 0.001*	-0.13	< 0.001*
**Residence** (ref: rural)	0.02	0.157	0.01	0.579	0.05	0.003*	0.05	0.002*	0.03	0.108	0.05	0.021*
**Home type** (ref: detached house)	0.09	< 0.001*	0.07	< 0.001*	0.08	< 0.001*	0.06	< 0.001*	0.08	< 0.001*	0.05	0.009*
**Homeownership** (ref: owner)	0.11	< 0.001*	0.10	< 0.001*	0.11	< 0.001*	0.10	< 0.001*	0.10	< 0.001*	0.11	< 0.001*
**Province**												
Alberta	-0.05	0.012*	0.01	0.474	0.03	0.082	0.11	< 0.001*	-0.06	0.019*	0.04	0.115
British Columbia	-0.12	0.316	-0.13	< 0.001*	0.12	0.334	-0.12	< 0.001*	0.15	< 0.001*	-0.03	0.167
Manitoba	0.03	0.074	0.06	0.001*	0.05	0.007*	0.08	< 0.001*	0.05	0.020*	0.05	0.041*
Newfoundland/ Labrador	0.05	0.006*	0.08	< 0.001*	0.02	0.249	0.03	0.104	-0.04	0.136	-0.06	0.010*
Nova Scotia	-0.09	< 0.001*	-0.08	< 0.001*	-0.12	< 0.001*	-0.08	< 0.001*	-0.06	0.014*	0.01	0.828
Ontario	-0.01	0.841	0.06	< 0.001*	0.01	0.946	0.02	0.362	-0.05	0.012*	0.04	0.085
Quebec	0.06	0.001*	0.04	0.040*	-0.01	0.526	0.02	0.228	-0.05	0.030*	-0.04	0.080
**Education‡** (ref: below secondary)	-0.08	< 0.001*	-0.08	0.001*	-0.06	0.011*	-0.09	< 0.001*	-0.05	0.081	-0.08	0.005*
**Occupation** (ref: manual)	-0.03	0.089	-0.04	0.040*	-0.03	0.367	-0.06	0.121	-0.003	0.935	-0.03	0.384
**Retirement** (ref: retired)	-0.06	< 0.001*	-0.06	< 0.001*	-0.06	0.001*	-0.05	0.004*	-0.07	< 0.001*	-0.06	0.002
**Total Household Income**												
Below $50,000	0.14	< 0.001*	0.15	< 0.001*	0.16	< 0.001*	0.19	< 0.001*	0.15	< 0.001*	0.12	< 0.001*
$50,000 - $99,999	-0.11	< 0.001*	-0.11	< 0.001*	-0.14	< 0.001*	-0.16	< 0.001*	-0.12	< 0.001*	-0.09	< 0.001*
$100,000 and above	-0.04	0.075	-0.07	0.001*	-0.04	0.067	-0.05	0.006*	-0.06	0.020*	-0.05	0.045*
**Wealth (Investments)**												
< $50,000	0.16	< 0.001*	0.14	< 0.001*	0.16	< 0.001*	0.17	< 0.001*	0.17	< 0.001*	0.15	< 0.001*
$50,000 - <$100,000	0.03	0.201	0.02	0.226	0.01	0.557	0.04	0.038*	0.01	0.676	0.03	0.211
$100,000 - <$1 M	-0.13	< 0.001*	-0.09	< 0.001*	-0.11	< 0.001*	-0.14	< 0.001*	-0.09	< 0.001*	-0.10	< 0.001*
$1 M and above	-0.03	0.118	-0.08	< 0.001*	-0.06	0.001*	-0.07	< 0.001*	-0.11	< 0.001*	-0.09	0.001*
**Social status†** (ref: unit increase)	-0.03	0.089	-0.09	< 0.001*	-0.04	0.055	-0.07	0.001*	-0.08	0.002*	-0.09	< 0.001*
**Religious affiliation** (ref: no)	0.04	0.051	0.07	< 0.001*	0.03	0.097	0.05	0.011*	0.02	0.355	0.03	0.221

Source: weighted Canadian Longitudinal Study on Ageing dataset. Standardised regression coefficient (β) = zero-order correlation coefficient (r). * = β was significant at *p* < 0.05. OECD = Organisation for Economic Cooperation and Development. FU1 = follow up (1) FU2 = follow up (2) TUG = timed-up and go test score (sec). 4MWT = four-meter walk test score (sec). † = FU2 analysis was completed with FU1 demographic variable. ‡ = FU1 and FU2 analysis was completed with baseline demographic variable

Table 2 shows the detailed cycle-wise bivariate association between mobility outcomes and modifiable sociodemographic factors. Specifically, marital status, type of home, home ownership, and total household income had a significant bivariate association with both TUG and 4MWT. Being married was associated with better performance on both tests (TUG β= -0.08, *p* < 0.001; 4MWT β= -0.09, *p* < 0.001). Living in an apartment compared to a detached house (TUG β = 0.09, *p* < 0.001; 4MWT β = 0.07, *p* < 0.001) and tenancy compared to being the owner of the house (TUG β = 0.11, *p* < 0.001; 4MWT β = 0.10, *p* < 0.001) were linked to higher mobility decline.

### Multivariate analysis

**Fig. 1:**
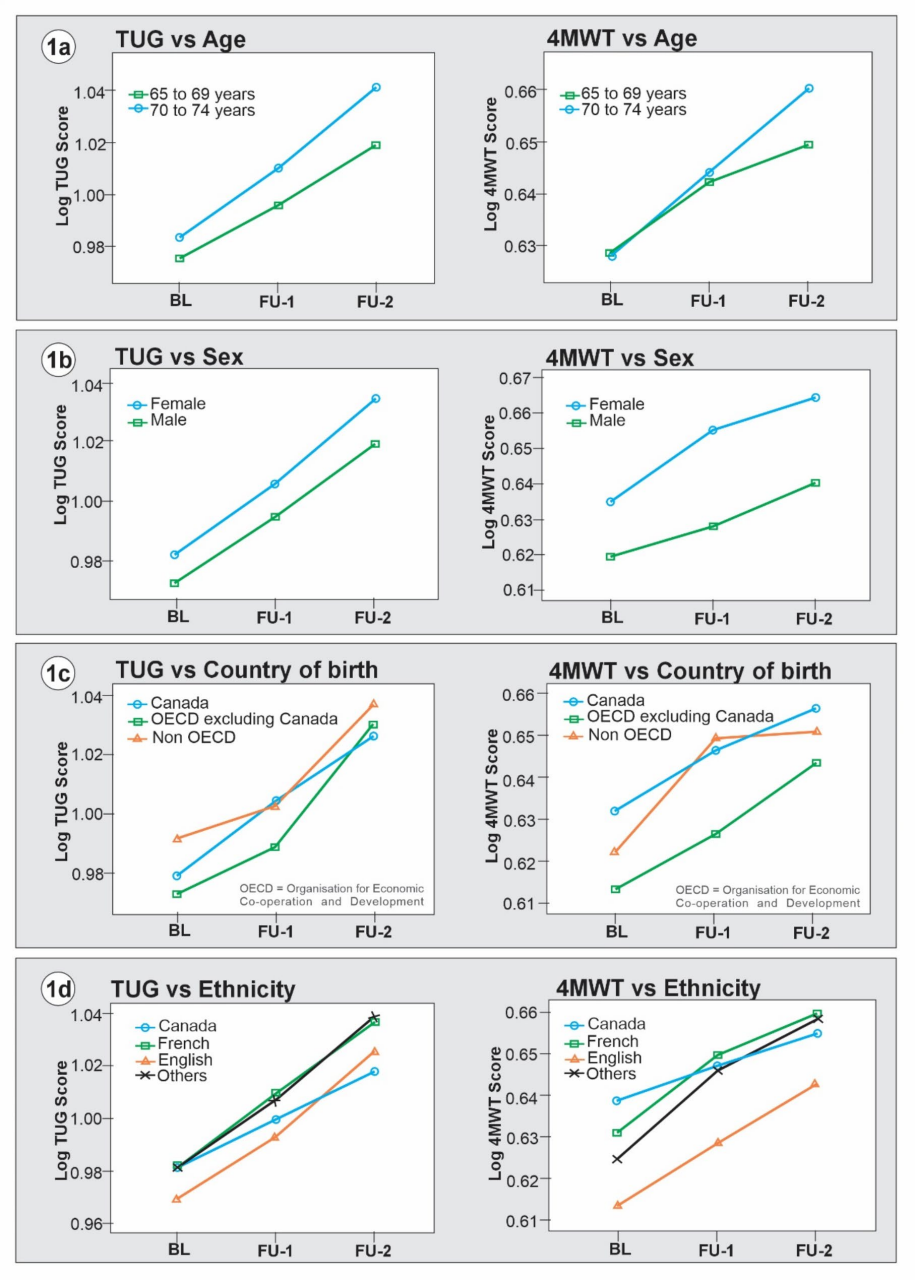
Marginal mean differences in mobility trajectory across sociodemographic factors: age, sex, country of birth and ethnicity. Source: weighted CLSA dataset

**Fig. 2:**
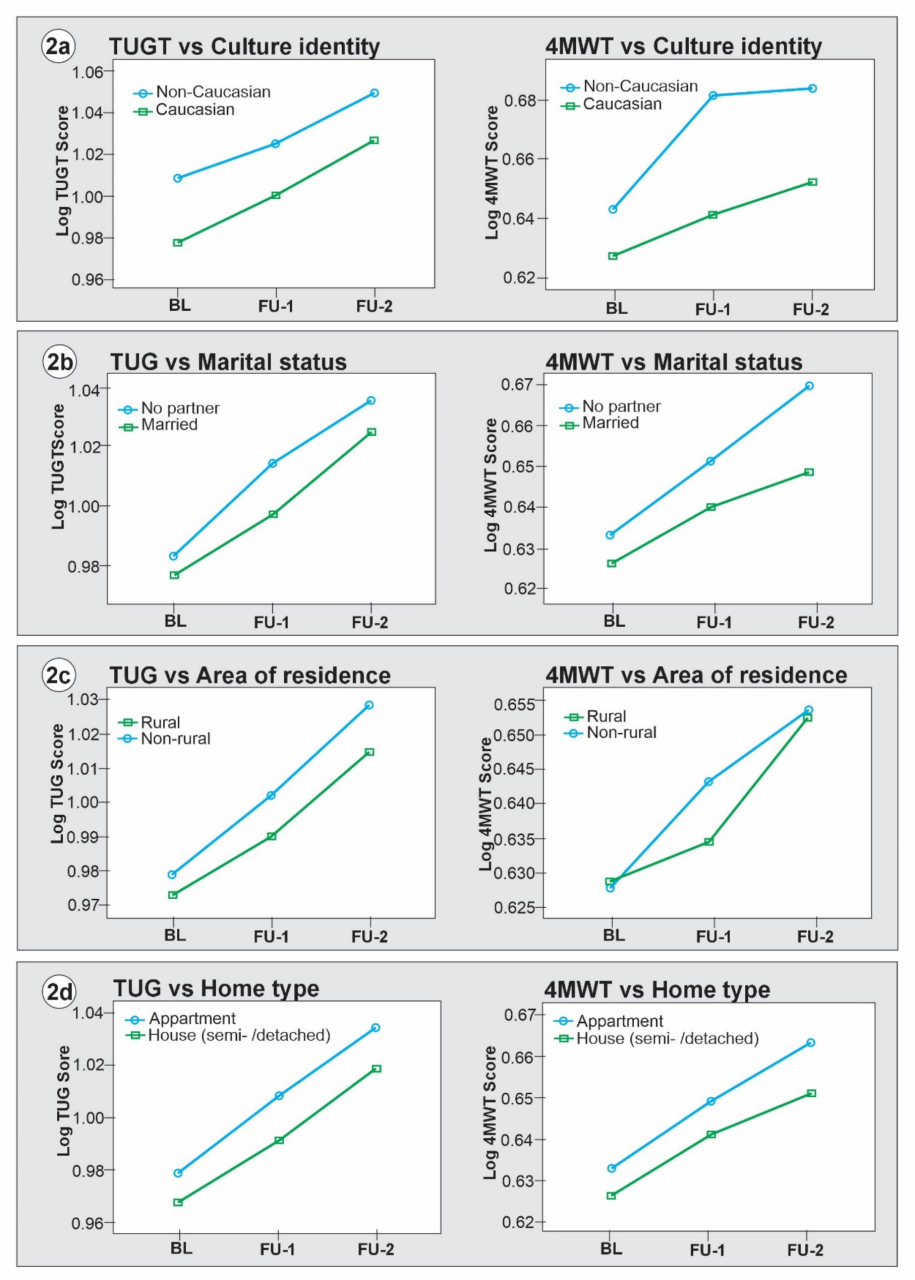
Marginal mean differences in mobility trajectory across sociodemographic factors: cultural identity, marital status, residence, and home type. Source: weighted CLSA dataset

**Fig. 3:**
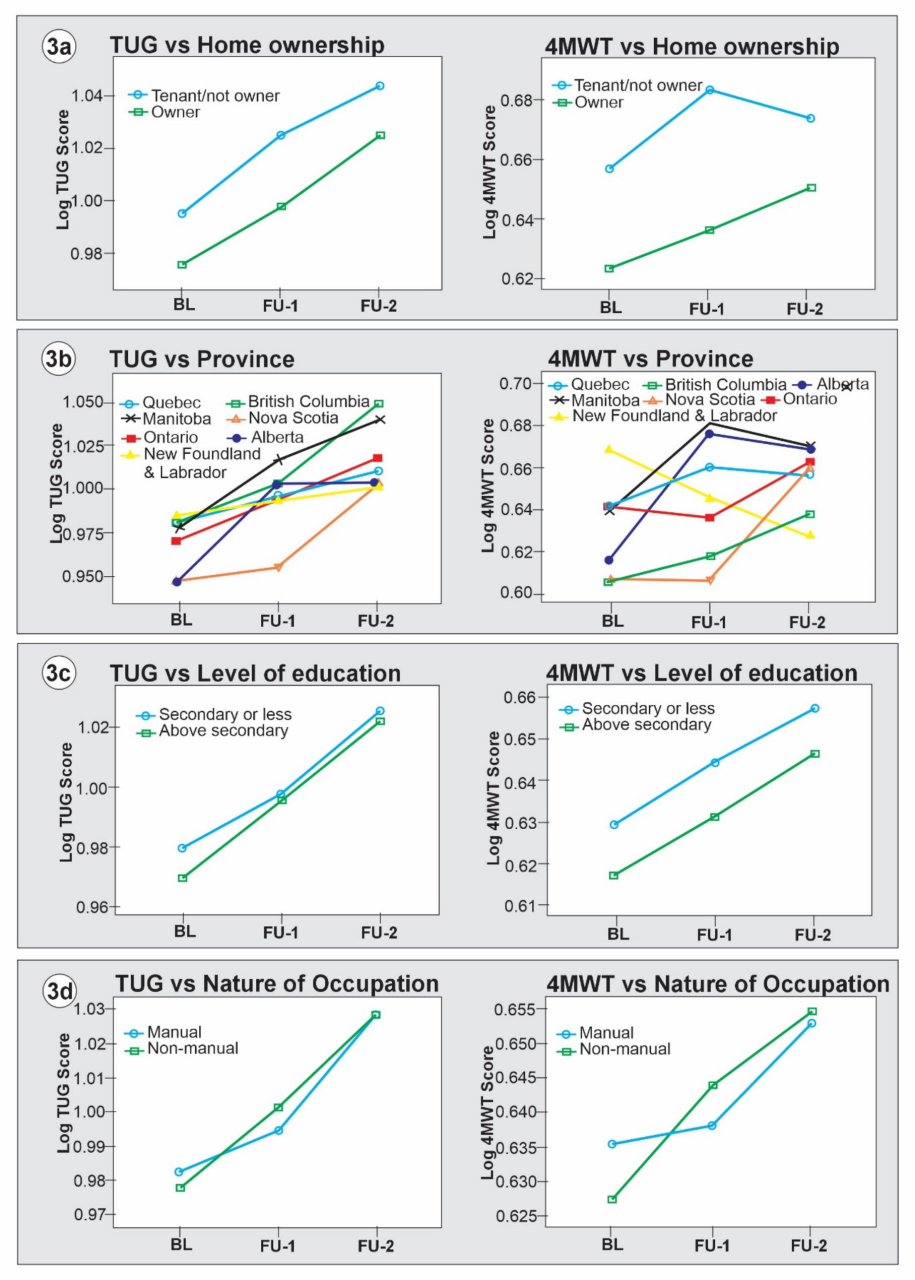
Marginal mean differences in mobility trajectory across sociodemographic factors: home ownership, province, education, and occupation. Source: weighted CLSA dataset

**Fig. 4:**
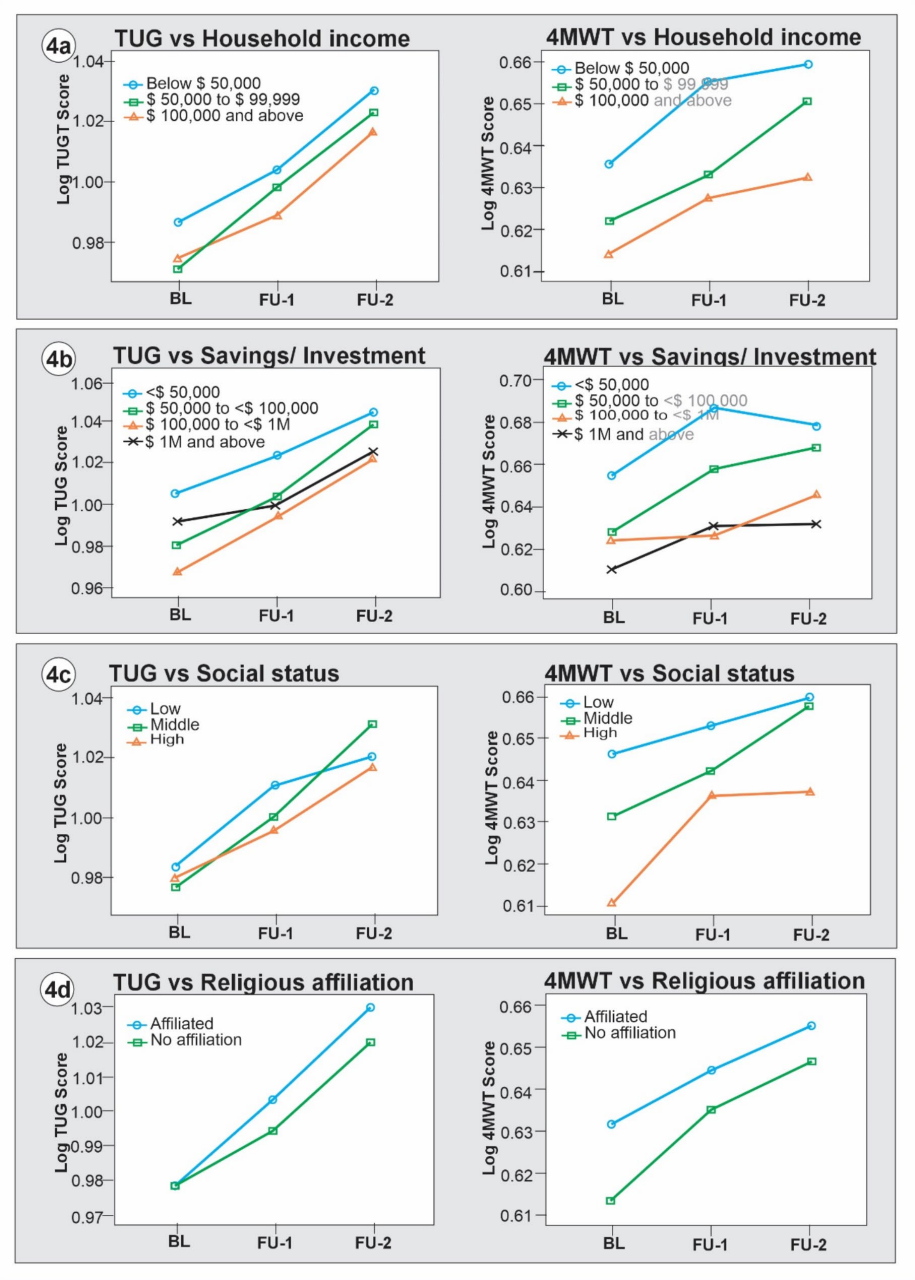
Marginal mean differences in mobility trajectory across sociodemographic factors: household income, wealth, social status, and religion. Source: weighted CLSA dataset

The mixed-design ANOVA results (Table 3) showed significant main effects of sociodemographic variables, study cycles, and significant sociodemographic*cycle interaction effects. However, there was no significant main effect of occupation type (TUG *F* [1, 27061] = 0.26, *p* = 0.611, ƞ^2^_p_ = 0.000; 4MWT *F* [1, 27146] = 0.01, *p* = 0.910, ƞ^2^_p_ = 0.000) and area of residence (4MWT *F* [1, 30364] = 3.36, *p* = 0.067, ƞ^2^_p_ = 0.000). The estimated marginal mean differences in *log* TUG and *log* 4MWT scores at baseline, FU1 and FU2 across the categories of the sociodemographic variables were plotted in Figs. 1, 2, 3 and 4.

**Table 3:** Mixed-design ANOVA for the effects of sociodemographic factors and study cycle on mobility decline

Parameter	TUG			4MWT		
	Partial Eta Squared (ƞ^2^_*p*_)	F-statistic (df)	*p*-value	Partial Eta Squared (ƞ^2^_*p*_)	F-statistic (df)	*p*-value
**Age group**	0.011	329.64 (1, 30277)	< 0.001*	0.001	24.02 (1, 30364)	< 0.001*
Cycle	0.184	6828.08 (2, 60554)	< 0.001*	0.046	1461.83 (2, 60728)	< 0.001*
Age group ^x^ Cycle	0.005	142.27 (2, 60554)	< 0.001*	0.003	76.46 (2, 60728)	< 0.001*
**Sex at birth**	0.007	207.12 (1, 30277)	< 0.001*	0.026	805.89 (1, 30364)	< 0.001*
Cycle	0.179	6599.49 (2, 60554)	< 0.001*	0.043	1350.08 (2, 60728)	< 0.001*
Sex ^x^ Cycle	0.001	22.43 (2, 60554)	< 0.001*	0.002	73.00 (2, 60728)	< 0.001*
**Country of birth**	0.002	25.58 (2, 30276)	< 0.001*	0.009	135.11 (2, 30363)	< 0.001*
Cycle	0.071	2297.81 (2, 60552)	< 0.001*	0.017	532.57 (2, 60726)	< 0.001*
Country of birth ^x^ Cycle	0.006	88.85 (4, 60552)	< 0.001*	0.001	16.11 (4, 60726)	< 0.001*
**Ethnicity**	0.007	66.96 (3, 30275)	< 0.001*	0.012	122.38 (3, 30362)	< 0.001*
Cycle	0.169	6142.55 (2, 60550)	< 0.001*	0.041	1302.60 (2, 60724)	< 0.001*
Ethnicity ^x^ Cycle	0.009	87.39 (6, 60550)	< 0.001*	0.004	43.84 (6, 60724)	< 0.001*
**Cultural identity**	0.005	156.46 (1, 30277)	< 0.001*	0.007	200.40 (1, 30364)	< 0.001*
Cycle	0.027	842.02 (2, 60554)	< 0.001*	0.012	375.35 (2, 60728)	< 0.001*
Cultural identity ^x^ Cycle	0.000	7.94 (2, 60554)	< 0.001*	0.002	49.10 (2, 60728)	< 0.001*
**Marital status**	0.005	140.32 (1, 30277)	< 0.001*	0.006	196.86 (1, 30364)	< 0.001*
Cycle	0.143	5043.87 (2, 60554)	< 0.001*	0.041	1310.07 (2, 60728)	< 0.001*
Marital status ^x^ Cycle	0.002	63.16 (2, 60554)	< 0.001*	0.003	79.16 (2, 60728)	< 0.001*
**Area of residence**	0.009	90.12 (3, 30275)	< 0.001*	0.000	3.36 (1, 30364)	0.067
Cycle	0.045	1412.01 (2, 60554)	< 0.001*	0.010	316.25 (2, 60728)	< 0.001*
Residence ^x^ Cycle	0.000	12.24 (2, 60554)	< 0.001*	0.000	13.35 (2, 60728)	< 0.001*
**Home type**	0.005	150.22 (1, 30163)	< 0.001*	0.003	77.75 (1, 30250)	< 0.001*
Cycle	0.124	4283.46 (2, 60326)	< 0.001*	0.032	987.75 (2, 60500)	< 0.001*
Home ^x^ Cycle	0.001	15.19 (2, 60326)	< 0.001*	0.000	12.73 (2, 60500)	< 0.001*
**House ownership**	0.011	330.83 (1, 30277)	< 0.001*	0.028	875.79 (1, 30364)	< 0.001*
Cycle	0.091	3028.39 (2, 60554)	< 0.001*	0.018	560.98 (2, 60728)	< 0.001*
House ownership ^x^ Cycle	0.001	29.73 (2, 60554)	< 0.001*	0.004	129.93 (2, 60728)	< 0.001*
**Province of residence**	0.022	112.55 (6, 30272)	< 0.001*	0.049	262.33 (6, 30359)	< 0.001*
Cycle	0.049	1543.57 (2, 60544)	< 0.001*	0.009	289.72 (2, 60718)	< 0.001*
Province ^x^ Cycle	0.042	219.88 (12, 60544)	< 0.001*	0.032	166.63 (12, 60718)	< 0.001*
**Education**	0.001	21.22 (1, 18614)	< 0.001*	0.007	127.23 (1, 18671)	< 0.001*
Cycle	0.161	3576.84 (2, 37228)	< 0.001*	0.048	939.74 (2, 37342)	< 0.001*
Education ^x^ Cycle	0.002	28.25 (2, 37228)	< 0.001*	0.000	1.98 (2, 37342)	0.139
**Occupation type**	0.000	0.26 (1, 27061)	0.611	0.000	0.01 (1, 27146)	0.910
Cycle	0.106	3205.28 (2, 54122)	< 0.001*	0.018	502.64 (2, 54292)	< 0.001*
Occupation ^x^ Cycle	0.002	45.53 (2, 54122)	< 0.001*	0.002	51.45 (2, 54292)	< 0.001*
**Retirement status**	0.008	250.60 (1, 30277)	< 0.001*	0.008	256.06 (1, 30364)	< 0.001*
Cycle	0.142	5011.10 (2, 60554)	< 0.001*	0.030	934.60 (2, 60728)	< 0.001*
Retirement ^x^ Cycle	0.002	63.48 (2, 60554)	< 0.001*	0.001	19.14 (2, 60728)	< 0.001*
**Total household income (THI)**	0.006	78.24 (2, 27911)	< 0.001*	0.016	222.28 (2, 27977)	< 0.001*
Cycle	0.090	2759.29 (2, 55822)	< 0.001*	0.019	543.70 (2, 55954)	< 0.001*
THI ^x^ Cycle	0.003	37.35 (4, 55822)	< 0.001*	0.003	47.64 (4, 55954)	< 0.001*
**Wealth/Investment**	0.029	259.81 (3, 26318)	< 0.001*	0.063	587.34 (3, 26391)	< 0.001*
Cycle	0.122	3655.30 (2, 52636)	< 0.001*	0.037	1009.75 (2, 52782)	< 0.001*
Wealth ^x^ Cycle	0.008	72.51 (6, 52636)	< 0.001*	0.015	135.87 (6, 52782)	< 0.001*
**Social status**	0.001	19.97 (2, 29623)	< 0.001*	0.014	204.40 (2, 29696)	< 0.001*
Cycle	0.092	2991.37 (2, 59246)	< 0.001*	0.021	626.29 (2, 59392)	< 0.001*
Social status ^x^ Cycle	0.008	123.62 (4, 59246)	< 0.001*	0.005	72.27 (4, 59392)	< 0.001*
**Religion affiliation**	0.001	38.64 (1, 30102)	< 0.001*	0.005	149.31 (1, 30189)	< 0.001*
Cycle	0.113	3835.85 (2, 60204)	< 0.001*	0.036	1114.68 (2, 60378)	< 0.001*
Religion ^x^ Cycle	0.002	50.47 (2, 60204)	< 0.001*	0.001	40.22 (2, 60378)	< 0.001*

Source: weighted Canadian Longitudinal Study on Ageing dataset. * = *F*-statistic was significant at *p* < 0.05. TUG = timed-up and go test score (sec). 4MWT = four-meter walk test score (sec)

The post hoc pairwise comparison of the *log mean differences* [*MD*] showed that people aged 70 to 74 years had a significant mobility decline compared to younger counterparts 65 to 69 years (TUG *MD* = 0.015, *p* < 0.001; 4MWT *MD* = 0.004, *p* < 0.001). Females had a significant mobility decline than males (TUG *MD* = 0.012, *p* < 0.001; 4MWT *MD* = 0.022, *p* < 0.001). Mobility decline trajectory of other non-modifiable factors, such as country of birth and ethnicity, are shown in Fig. 1.

Figures 2, 3 and 4 show the linear trend of mobility decline across categories of modifiable sociodemographic factors. Specifically, Fig. 2 shows that non-Caucasians had a significant mobility decline relative to Caucasians (TUG *MD* = 0.026, *p* < 0.001; 4MWT *MD* = 0.029, *p* < 0.001). Participants who were single/divorced/separated/widowed had a significant mobility decline compared to their married counterparts (TUG *MD* = 0.011, *p* < 0.001; 4MWT *MD* = 0.013, *p* < 0.001). Non-rural dwellers had a significant mobility decline compared to rural-dwelling older adults (TUG *MD* = 0.011, *p* < 0.001). The 4MWT *MD* = 0.003 (*p* = 0.067) was not significant. Older adults who lived in apartments or condominiums had a significant mobility decline relative to those who lived in a single detached, semi-detached, duplex or townhouse (TUG *MD* = 0.013, *p* < 0.001; 4MWT *MD* = 0.009, *p* < 0.001).

Figure 3 shows that older adults who were tenants had a significant mobility decline than those who owned their homes (TUG *MD* = 0.022, *p* < 0.001; 4MWT *MD* = 0.034, *p* < 0.001). Older adults with secondary education or less had a significant mobility decline than their counterparts with higher education levels (TUG *MD* = 0.005, *p* < 0.001; 4MWT *MD* = 0.012, *p* < 0.001). However, there was no significant difference in mobility decline between participants who engaged in manual vs. non-manual occupations (TUG *MD*=-0.01, *p* = 0.611; 4MWT *MD* = 0.000, *p* = 0.910). Figure 3 (b) shows the mobility trajectory across the seven Canadian provinces included in CLSA. Briefly, the mean *log* TUG and 4MWT showed that older adults in Manitoba had the highest rate of mobility decline. The TUG showed that Nova Scotia had the lowest decline rate, while the 4MWT suggests that British Columbia had the lowest decline, followed closely by Nova Scotia.

Figure 4 shows that older adults in low-income households had a significant mobility decline than those in the middle (TUG *MD* = 0.010, *p* < 0.001; 4MWT *MD* = 0.015, *p* < 0.001) and high-income households (TUG *MD* = 0.014, *p* < 0.001; 4MWT *MD* = 0.025, *p* < 0.001). Participants with moderate wealth *($100*,*000 - $999*,*999)* had better mobility trajectory than those less than *$50*,*000* (TUG *MD*=-0.030, *p* < 0.001; 4MWT *MD*=-0.041, *p* < 0.001), and *$50*,*000 - $100*,*000* (TUG *MD*=-0.013, *p* < 0.001; 4MWT *MD*=-0.019, *p* < 0.001). However, participants with moderate wealth *($100*,*000 - $999*,*999)* had better mobility than those with *$1*,*000*,*000 and above* (TUG *MD*=-0.012, *p* < 0.001; 4MWT *MD*=-0.008, *p* < 0.001). Older adults of lower social status had a significant mobility decline than those in the middle (TUG *MD* = 0.002, *p* = 0.112; 4MWT *MD* = 0.009, *p* < 0.001) and high social status (TUG *MD* = 0.007, *p* < 0.001; 4MWT *MD* = 0.025, *p* < 0.001). Participants with religious affiliation had a more significant mobility decline than their irreligious counterparts (TUG *MD* = 0.006, *p* < 0.001; 4MWT *MD* = 0.012, *p* < 0.001). Retirees’ mobility declined significantly more than their counterparts who were partly or not retired (TUG *MD* = 0.015, *p* < 0.001; 4MWT *MD* = 0.014, *p* < 0.001).

We completed a simultaneous entry multivariate linear regression to estimate the association between sociodemographic factors and mobility outcome at baseline and mobility decline at FU1 and FU2. Tables 4 and 5 show the results for TUG and 4MWT, respectively. Sociodemographic determinants of mobility decline after the 6-year follow-up (FU2 minus baseline) were reported in the text if they had a significant standardised regression coefficient (β) and the same effect direction for both TUG and 4MWT. For simplicity, only the results for TUG were presented in the text.

**Table 4:** Multivariate longitudinal regression for association between TUG and sociodemographic factors

Variable	Baseline (BL)		FU1 minus BL		FU2 minus FU1		FU2 minus BL	
***Baseline demographic characteristics***	β-statistic	*p*-value	β-statistic	*p*-value	β-statistic	*p*-value	β-statistic	*p*-value
**Age** (increase in years)	0.100	< 0.001*	0.107	< 0.001*	0.036	< 0.001*	0.088	< 0.001*
**Sex** (ref: female)	-0.025	< 0.001*	0.026	< 0.001*	-0.027	< 0.001*	-0.035	< 0.001*
**Marital status** (ref: has no partner)	-0.001	0.906	-0.027	< 0.001*	-0.014	0.060	-0.057	< 0.001*
**Country of birth** (ref: Canada)								
OECD excluding Canada	0.014	0.052	-0.026	< 0.001*	0.013	0.054	-0.046	< 0.001*
Non-OECD	-0.031	< 0.001*	-0.063	< 0.001*	-0.085	< 0.001*	-0.083	< 0.001*
**Ethnicity** (ref: Canada)								
French	0.0035	< 0.001*	-0.017	0.004*	0.038	< 0.001*	0.013	0.101
English	-0.028	0.001*	-0.023	0.001*	-0.001	0.920	-0.006	0.560
Others	-0.047	< 0.001*	-0.025	0.001*	0.005	0.583	-0.003	0.720
**Cultural identity** (ref: non-Caucasian)	-0.036	< 0.001*	-0.076	< 0.001*	-0.047	< 0.001*	-0.063	< 0.001*
**Province** (ref: Ontario)								
Alberta	-0.026	< 0.001*	0.077	< 0.001*	-0.051	< 0.001*	0.035	< 0.001*
British Columbia	-0.004	0.637	0.062	< 0.001*	0.130	< 0.001*	0.169	< 0.001*
Manitoba	0.022	0.002*	0.047	< 0.001*	0.007	0.332	0.025	0.001*
Newfoundland/ Labrador	0.011	0.102	0.006	0.291	-0.010	0.116	-0.008	0.269
Nova Scotia	-0.044	< 0.001*	-0.026	< 0.001*	0.001	0.882	-0.014	0.053
Quebec	-0.030	0.003*	-0.044	< 0.001*	-0.108	< 0.001*	-0.106	< 0.001*
**Area of residence** (ref: rural)	0.020	0.002*	0.011	0.078	-0.015	0.063	-0.010	0.264
**House** (ref: semi-/detached house)	0.029	< 0.001*	0.016	0.093	-0.027	0.002*	0.028	0.018*
**House ownership** (ref: owner)	0.000	1.000	-0.010	0.112	-0.035	< 0.001*	0.050	< 0.001*
**THI $** (ref: below 50,000)								
50,000–99,999	-0.031	< 0.001*	0.011	0.106	-0.006	0.452	0.039	< 0.001*
100,000 and above	-0.018	0.024*	-0.011	0.103	0.040	< 0.001*	0.006	0.466
**Wealth/Invest. $** (ref: below 50,000)								
50,000–100,000	-0.045	< 0.001*	-0.054	< 0.001*	-0.038	< 0.001*	-0.089	< 0.001*
100,000–1,000,000	-0.098	< 0.001*	-0.078	< 0.001*	-0.043	< 0.001*	-0.084	< 0.001*
1,000,000 and above	-0.071	< 0.001*	-0.069	< 0.001*	-0.021	0.024*	-0.039	< 0.001*
**Education** (ref: secondary and below)	-0.022	0.001*	0.062	< 0.001*	0.021	0.002*	0.043	< 0.001*
**Occupation** (ref: manual)	-0.055	< 0.001*	-0.028	< 0.001*	-0.013	0.044*	-0.002	0.833
**Retirement** (ref: retired)	-0.049	< 0.001*	-0.035	< 0.001*	-0.053	< 0.001*	-0.058	< 0.001*
**Social status ladder** (unit increase)	0.049	< 0.001*	0.011	0.079	-0.015	0.023*	-0.018	0.025*
**Religion affiliation** (ref: no)	-0.014	0.035*	0.011	0.045*	0.025	< 0.001*	0.036	< 0.001*
**Smoked 100 cigarettes in life** (ref: no)	0.090	< 0.001*	0.047	< 0.001*	-0.009	0.163	0.002	0.750
**Frequency of alcohol** (a unit increase)	-0.100	< 0.001*	-0.014	0.015*	-0.025	< 0.001*	-0.028	< 0.001*
**Chronic disease status** (ref: no)	-0.007	0.285	0.035	< 0.001*	-0.005	0.480	0.024	0.001*
**General health rating** (ref: poor)	-0.188	< 0.001*	-0.099	< 0.001*	-0.136	< 0.001*	-0.164	< 0.001*
***Sociodemographic change***								
**Marital status change** (ref: no partner)			0.063	< 0.001*	0.025	< 0.001*	-0.048	< 0.001*
**Social status ladder** (unit increase)			-0.035	< 0.001*	-	-	-0.026	0.001*
**More religious** (ref: same state)			0.014	0.009*	-0.032	< 0.001*	0.017	0.029*
**Less religious** (ref: same state)			-0.040	< 0.001*	0.016	0.014*	-0.002	0.768
**Education gain** (ref: no)			-0.007	0.222	-0.006	0.317	-0.038	< 0.001*
**Remained retired** (ref: no)			0.045	< 0.001*	0.032	< 0.001*	0.084	< 0.001*
**House** (ref: semi-/detached house)			0.039	< 0.001*	0.074	< 0.001*	-0.144	< 0.001*
**Change of residence** (ref: went rural)			-0.010	0.120	-0.034	< 0.001*	0.035	0.036*
***F-statistics***	*F* (32, 22664) = 82.61, *p* < 0.001*		*F* (40, 20690) = 381.99, *p* < 0.001*		*F* (39, 13253) = 37.44, *p* < 0.001*		*F* (40, 12424) = 181.42, *p* < 0.001*	
***R-statistics***	*R* = 0.32, AR^2^ = 0.10		*R* = 0.66, AR^2^ = 0.43		*R* = 0.70, AR^2^ = 0.49		*R* = 0.64, AR^2^ = 0.41	

Source: weighted Canadian Longitudinal Study on Ageing dataset. * = standardised regression coefficient (β) was significant at *p* < 0.05. THI = total household income. OECD = Organisation for Economic Cooperation and Development. R = correlation coefficient. AR^2^ = adjusted R squared

**Table 5:** Multivariate longitudinal regression for association between 4MWT and sociodemographic factors

Variable	Baseline (BL)		FU1 minus BL		FU2 minus FU1		FU2 minus BL	
***Baseline demographic characteristics***	β-statistic	*p*-value	β-statistic	*p*-value	β-statistic	*p*-value	β-statistic	*p*-value
**Age** (increase in years)	0.069	< 0.001*	0.085	< 0.001*	0.074	< 0.001*	0.092	< 0.001*
**Sex** (ref: female)	-0.083	< 0.001*	-0.070	< 0.001*	-0.059	< 0.001*	-0.066	< 0.001*
**Marital status** (has no partner)	0.016	0.029*	0.029	< 0.001*	-0.147	< 0.001*	-0.171	< 0.001*
**Country of birth** (ref: Canada)								
OECD excluding Canada	0.024	0.001*	-0.032	< 0.001*	-0.032	< 0.001*	-0.064	< 0.001*
Non-OECD	-0.035	< 0.001*	-0.052	< 0.001*	-0.033	0.001*	-0.041	< 0.001*
**Ethnicity** (ref: Canada)								
French	0.052	< 0.001*	0.055	< 0.001*	-0.041	< 0.001*	-0.026	0.003*
English	-0.022	0.010*	-0.003	0.739	-0.029	0.003*	-0.012	0.241
Others	-0.044	< 0.001*	0.030	< 0.001*	0.012	0.237	0.050	< 0.001*
**Cultural identity** (ref: non-Caucasian)	-0.018	0.020*	-0.072	< 0.001*	-0.022	0.020*	-0.048	< 0.001*
**Province** (ref: Ontario)								
Alberta	0.013	0.076	0.100	< 0.001*	0.016	0.054	0.071	< 0.001*
British Columbia	-0.144	< 0.001*	-0.084	< 0.001*	-0.055	< 0.001*	-0.087	< 0.001*
Manitoba	0.046	< 0.001*	0.060	< 0.001*	-0.058	< 0.001*	-0.027	0.002*
Newfoundland/ Labrador	0.013	0.044*	-0.016	0.006*	-0.045	< 0.001*	-0.054	< 0.001*
Nova Scotia	-0.041	< 0.001*	-0.017	0.004*	0.013	0.099	0.005	0.562
Quebec	0.002	0.868	-0.046	< 0.001*	-0.121	< 0.001*	-0.082	< 0.001*
**Area of residence** (ref: rural)	0.013	0.041*	0.034	< 0.001*	-0.036	< 0.001*	-0.014	0.151
**House** (ref: semi-/detached house)	0.058	< 0.001*	0.002	0.849	-0.036	< 0.001*	0.008	0.525
**House ownership** (ref: owner)	-0.030	< 0.001*	-0.007	0.319	-0.005	0.585	0.023	0.013*
**THI $** (ref: below 50,000)								
50,000–99,999	0.011	0.159	-0.052	< 0.001*	0.035	< 0.001*	0.041	< 0.001*
100,000 and above	0.013	0.108	-0.010	0.175	-0.011	0.206	-0.040	< 0.001*
**Wealth/Invest. $** (ref: below 50,000)								
50,000–100,000	-0.075	< 0.001*	-0.079	< 0.001*	-0.047	< 0.001*	-0.074	< 0.001*
100,000–1,000,000	-0.128	< 0.001*	-0.139	< 0.001*	-0.065	< 0.001*	-0.088	< 0.001*
1,000,000 and above	-0.112	< 0.001*	-0.116	< 0.001*	-0.030	0.004*	-0.033	0.003*
**Education** (ref: secondary and below)	-0.029	< 0.001*	0.006	0.282	0.011	0.149	0.005	0.562
**Occupation** (ref: manual)	-0.046	< 0.001*	0.015	0.009*	0.001	0.937	0.016	0.039*
**Retirement** (ref: retired)	-0.061	< 0.001*	-0.045	< 0.001*	-0.010	0.257	-0.040	< 0.001*
**Social status ladder** (unit increase)	0.032	< 0.001*	0.035	< 0.001*	0.003	0.681	0.033	< 0.001*
**Religion affiliation** (ref: no)	-0.013	0.049*	-0.055	< 0.001*	0.065	< 0.001*	0.037	< 0.001*
**Smoked 100 cigarettes in life** (ref: no)	0.054	< 0.001*	0.052	< 0.001*	0.007	0.317	0.053	< 0.001*
**Frequency of alcohol** (a unit increase)	-0.085	< 0.001*	-0.035	< 0.001*	-0.030	< 0.001*	-0.025	0.003*
**Chronic disease status** (ref: no)	0.010	0.117	-0.005	0.412	0.032	< 0.001*	0.000	0.998
**General health rating** (ref: poor)	-0.153	< 0.001*	-0.097	< 0.001*	-0.083	< 0.001*	-0.120	< 0.001*
***Sociodemographic change***								
**Marital status change** (ref: no)			0.046	< 0.001*	0.013	0.081	0.029	< 0.001*
**Social status ladder** (unit increase)			-0.010	0.124	-	-	-0.075	< 0.001*
**More religious** (ref: same state)			-0.049	< 0.001*	-0.053	< 0.001*	0.002	0.835
**Less religious** (ref: same state)			-0.028	< 0.001*	0.020	0.007*	-0.051	< 0.001*
**Education gain** (ref: no)			0.023	< 0.001*	0.004	0.560	0.010	0.184
**Remained retired** (ref: no)			0.067	< 0.001*	0.008	0.316	0.023	0.050*
**House** (ref: semi-/detached house)			0.037	< 0.001*	0.030	0.002*	-0.123	< 0.001*
**Change of residence** (ref: went rural)			-0.032	< 0.001*	0.011	0.209	0.003	0.866
***F-statistics***	*F* (32, 22615) = 91.11, *p* < 0.001*		*F* (40, 20674) = 260.77, *p* < 0.001*		*F* (39, 13300) = 169.86, *p* < 0.001*		*F* (40, 12435) = 112.38, *p* < 0.001*	
***R-statistics***	*R* = 0.34, AR^2^ = 0.11		*R* = 0.58, AR^2^ = 0.34		*R* = 0.58, AR^2^ = 0.34		*R* = 0.55, AR^2^ = 0.30	

Source: weighted Canadian Longitudinal Study on Ageing dataset. * = standardised regression coefficient (β) was significant at *p* < 0.05. THI = total household income. OECD = Organisation for Economic Cooperation and Development. R = correlation coefficient. AR^2^ = adjusted R squared

Increasing age (β = 0.088, *p* < 0.001), being a female (β=-0.035, *p* < 0.001), being born in Canada compared with other OECD (β=-0.046, *p* < 0.001) or non-OECD countries (β=-0.083, *p* < 0.001), and being a non-Caucasian (β=-0.063, *p* < 0.001) significantly associated with higher mobility decline rate.

The following modifiable factors were significant predictors of mobility decline. Economic factors such as having a THI of $50,000-$99,999 (β = 0.039, *p* < 0.001), wealth/investment worth lower than $50,000 compared to $50,000-$100,000 (β=-0.089, *p* < 0.001), $100,000-$1 M (β=-0.084, *p* < 0.001), $1 M and above (β=-0.039, *p* < 0.001). Employment factors include being retired at baseline (β=-0.058, *p* < 0.001) and remaining in retirement at FU2 (β = 0.084, *p* < 0.001). Residential factors such as living in Alberta (β = 0.035, *p* < 0.001) compared with Ontario, or in Ontario compared with Quebec (β=-0.106, *p* < 0.001), being a tenant (β = 0.050, *p* < 0.001), and remaining in a semi-/detached house up until FU2 (β=-0.144, *p* < 0.001).

Other modifiable variables include sociobehavioural factors such as not being married or not having a partner (β=-0.057, *p* < 0.001), having religious affiliation (β = 0.036, *p* < 0.001), low social status (β=-0.018, *p* = 0.025), a further drop in social status (β=-0.026, *p* < 0.001), lesser alcohol intake (β=-0.028, *p* < 0.001), and poor self-reported general health (β=-0.164, *p* < 0.001). The TUG (*F* [40, 12424] = 181.42, *p* < 0.001, *R* = 0.64, adjusted *R*^2^ = 0.41) and 4MWT models (*F* [40, 12435] = 112.38, *p* < 0.001, *R* = 0.55, adjusted *R*^2^ = 0.30) were robust, accounting for 41% and 30% of the total variance, respectively.

## Discussion

This longitudinal analysis provides valuable insights into the sociodemographic determinants of mobility decline among older adults in Canada. The findings underscore the complexity of ageing, highlighting the role of various sociodemographic factors in mobility decline. As evidenced by the significant associations found between mobility outcomes and age, gender, marital status, country of birth, cultural identity, province of residence, income, wealth, home ownership, dwelling type, religious affiliation, retirement, and social status, our study contributes to the growing body of literature on geriatric health and mobility [5, 7, 12, 17]. The weighted sociodemographic profile of the current study was similar to the original CLSA baseline comprehensive cohort [23] and other profiles of the Canadian older adult population [33]. The remainder of this section discusses non-modifiable factors, followed by modifiable factors, the concurrent validity of the outcome measures, and recommendations.

Non-modifiable sociodemographic determinants of older adults’ mobility trajectory identified in our study, such as age, sex, country of birth, ethnicity, and cultural identity, are consistent with existing literature. Similar to our result, Wu and Zhao [34] revealed that increasing age was associated with a decline in walking speed among older Chinese. A meta-analysis of age effect on walking mechanics showed age-related gait decline [35]. Our findings also align with previous research conducted among older adults from the USA, Taiwan, Korea, Mexico, China, Indonesia, and Bolivia, indicating that women often experience higher rates of mobility decline [36]. A 7-year follow-up longitudinal study found a significant sex difference in the prevalence of mobility disability among 10,263 community-dwelling older adults in the United States [37]. Beyond biological sex or physiological factors, socioculturally constructed gender roles may affect mobility outcomes in older women [38]. Life course accumulation of these roles, including childbearing, childcare, home-making, and other gendered economic activities such as food processing, may lead to earlier and more severe mobility disability in women.

Intersectionality of age, gender, and race exacerbates mobility decline in racialised older women [9, 34, 35, 37, 39]. For instance, while a younger Caucasian woman may experience gender discrimination, an older Black woman faces compounded discrimination due to ageism, sexism, and racism, creating additional structural disadvantages [40]. Among people of similar age and sex, non-Caucasians had a higher mobility decline rate than Caucasians [40, 41]. As Webber et al. [5] highlighted, demographic, social, and economic factors acting independently and cumulatively influence individuals’ experiences, opportunities, and behaviours leading to disproportionate mobility outcomes. To be effective, the policy action on sociodemographic determinants of health should be comprehensive and holistic [42, 43]. The idea that modifying gender roles can ameliorate mobility decline among older females extends to other non-genetically determined but socially construed non-modifiable sociodemographic factors, such as ethnicity and cultural identities. These factors can be socially engineered through a cultural practices review [41, 44], good governance, intentional equity, and social justice [43, 45–47].

Modifiable factors identified in our study were marital status, province of residence, housing, retirement, health condition, and economic factors such as income and savings. These factors are considered modifiable because they can be influenced by personal choices or targeted government policies. Being married or having a partner may improve the mobility trajectory of older adults [48, 49]. Hossain et al. [50] posited that married older adults had a lesser risk of mobility difficulty, whereas unmarried status was disadvantageous, particularly for women. It underscores that having aid in the house may not offer the positive effect of a partner. Married individuals have more household income, access to care, housing, and social support, which can help them maintain physical activity and engagement in daily activities [50]. Older adults who have lost their spouse may be encouraged to get a partner, cohabit, or coreside for companionship.

Residence in a province is a personal choice based on economic or environmental interests. A systematic review of provincial policies on ageing across Canada showed some critical differences [51]. The policies have implications for housing, post-retirement employment, income, and access to health. For instance, Alberta’s policy promised financial security, housing, and health care. While having similar statements, Nova Scotia specified workplace support to encourage older workers’ participation and post-retirement volunteering [51].

Our findings align with other studies that identify housing [52–54] and financial security [9, 55] as essential predictors of mobility outcomes in older adults. Higher-income or wealthy individuals have more education and high-paying but sedentary occupations [56–58], leading to inactivity and its life course sequelae. Conversely, low-income earners may have lesser education, manual jobs, limited health access, and early biophysical decline [59–61]. We found middle-income earners and moderately wealthy older adults to have better mobility than people at both extremes.

Aside from the study’s primary objective, we tested the validity of 4MWT using the TUG as a criterion. Pearson correlation analysis showed a strong positive longitudinal concurrent correlation between TUG and 4MWT scores. However, the lateral displacement of the curves in Figs. 1, 2, 3 and 4 showed that TUG is a more stable measure of mobility in this population compared to the 4MWT. Previous studies have established good psychometric and clinimetric properties of TUG among community-dwelling older adults [6, 28, 29]. While 4MWT has acceptable levels of validity and reliability among older adults [25], there is a paucity of data on its other psychometrics, such as normative values, responsiveness, and minimal clinically important changes [62].

The results and discussions of this study have highlighted areas for policy action. Ageing policies should be formed and implemented in cognisance of sociodemographic inequalities. National resources, infrastructures, utilities, and services should be distributed considering older adults, particularly those from lower socioeconomic backgrounds and women. Future research may investigate the role of technological advancements and their potential to mitigate the decline in mobility.

### Limitations

This secondary analysis has some limitations inherent in the CLSA comprehensive cohort. Data collection was limited to predominantly urban areas across seven of the ten Canadian provinces. Due to self-selection, cohort studies like CLSA, which require written consent, language proficiency, and in-person visits, may under-represent less literate, recent migrants, and those with health issues [23]. However, the weighted baseline variables of CLSA were comparable with estimates generated from Canadian census data and other nationally representative surveys [23]. One of the typical limitations of longitudinal studies is attrition or loss of follow-up. There were some missing data and variables and losses to follow up in FU1 and FU2, which was more remarkable in FU2 due to the COVID-19 pandemic. Our study may not have covered all the sociodemographic determinants of mobility decline and their intersectionality.

## Conclusion

This study highlights the impact of sociodemographic factors on the mobility trajectory of older adults. Non-modifiable factors such as being older, a woman, and non-Caucasian, along with modifiable factors including being a retiree, Canadian born, tenant, having no spouse or partner, lower income, social status, wealth and education, and the province of residence were significant predictors of mobility decline in the six-year follow-up multivariate model. These findings underscore the need for nuanced ageing policies that address both sociodemographic inequalities and economic disparities, ensuring equitable distribution of resources, particularly for vulnerable groups such as older women, non-Caucasians, and those with lower socioeconomic status. While the study acknowledges limitations, it offers crucial insights for future research and policy initiatives aimed at mitigating mobility decline among older adults.

## Data Availability

The data are available from the Canadian Longitudinal Study on Aging (https://www.clsa-elcv.ca/data-access) for researchers who meet the criteria for access to de-identified CLSA data. The datasets used in the present study were Baseline Comprehensive Dataset (version 7.0), Follow-up 1 Comprehensive Dataset (version 4.0), and Follow-up 2 Comprehensive Dataset (version 2.0).

## Abbreviations

4MWT: 4-metre walk test
ANOVA: Analysis of variance
BL: Baseline
CLSA: Canadian Longitudinal Study on Ageing
COVID-19: Coronavirus diseases of 2019
FU1: Follow up 1
FU2: Follow up 2
Log: Logarithm
THI: Total household income
TUG: Timed-up and go test
